# When to become an electronic business venture after the COVID-19 pandemic? The role of strategic orientation and perceived environmental turbulence in determining online market entry timing

**DOI:** 10.3389/fpubh.2022.989264

**Published:** 2022-09-13

**Authors:** Hongyi Mao, Changqing He, Xing Huang, Banggang Wu, Zhi Chen, Liying Zhou

**Affiliations:** ^1^School of Business Administration, Guizhou University of Finance and Economics, Guiyang, China; ^2^College of Economics and Management, Nanjing University of Aeronautics and Astronautics, Nanjing, China; ^3^Portsmouth Business School, University of Portsmouth, Portsmouth, United Kingdom; ^4^Business School, Sichuan Univeristy, Chengdu, China; ^5^School of Business Administration, Shanghai Lixin University of Accounting and Finance, Shanghai, China; ^6^Guizhou Key Laboratory of Big Data Statistical Analysis, Guiyang, China

**Keywords:** Guanxi orientation, entrepreneurial orientation, perceived environmental turbulence, entry timing, COVID-19 pandemic

## Abstract

After the COVID-19 epidemic, a growing number of commercial entities have decided to enter the online platform and operated as an electronic business venture. However, the timing of entering the online market is a strategically important issue. On the basis of social capital theory and resource-based view, this study attempts to understand the different impacts of two strategic orientations (i.e., Guanxi orientation and entrepreneurial orientation) and perceived environmental turbulence (i.e., market turbulence and political turbulence) on online market entry timing. We test four hypotheses using data collected from 174 Chinese companies. Our results confirm that entrepreneurial orientation negatively impacts online market entry timing, and this effect is moderated by perceived market turbulence such that the negative relationship between entrepreneurial orientation and online market entry timing will be strengthened in higher market turbulence. By contrast, Guanxi orientation positively impacts online market entry timing, and the positive relationship between Guanxi orientation and online market entry timing will be weakened in higher political turbulence. Implications and future research directions are discussed.

## Introduction

The COVID-19 pandemic has impacted some industries, but it has also brought significant opportunities to new industries and new business models (1–3). According to the United Nations Conference on Trade and Development, despite the easing of restrictions in many countries, e-commerce activities have been largely fueled by the pandemic, resulting in a marked online sales increase (4). In addition, a McKinsey report states that 20–30% of businesses moved online during the peak of the pandemic (5). After the COVID-19 epidemic, a growing number of commercial entities have decided to enter the online platform and operate as an electronic business venture (EBV). For those companies, the timing of entering the online market is strategically important (6). Most past studies on market entry timing have focused on traditional offline markets and have not considered the turbulence in the business environment brought about by the COVID-19 pandemic (7). However, the factors and mechanisms that influence the timing of becoming an EBV after the COVID-19 pandemic remain unclear.

The choice of when to enter a market is a critical strategic decision, which is greatly influenced by the company's strategic orientation (8, 9). Appropriate entry timing will bring companies with a competitive advantage in resources, conditions, and mechanisms (10). Social capital theory and resource-based view point out that corporate performance is closely related to its relationship network and its own capabilities (11). On the one hand, EBVs will strive to obtain the convenience of resources, policies, and information by establishing links with the outside world, and promoting economic transactions (12). On the other hand, they will focus on their own capacity building, taking advantage of market opportunities by exerting autonomy and innovation (13). These two behaviors reflect two different strategic orientations—Guanxi orientation and entrepreneurial orientation, which correspond to different corporate resource investment and allocation tendencies (14, 15), thus affecting the timing of companies entering the market.

Guanxi orientation is a key factor in building organizational external connections in the context of Chinese EBVs (6, 16). Many scholars believe that Guanxi orientation is an important guiding principle in decision-making, and Guanxi-oriented companies value the establishment and maintenance of personal relationships and tend to achieve business objectives through managerial ties with business partners (6). In the Chinese context, most Chinese companies leverage Guanxi activities for sharing resources, reducing risks, thereby improving the efficiency and effectiveness of business activities (17). However, whether Guanxi orientation will influence enterprises' market entry timing in the online market after the COVID-19 pandemic is an underexplored research topic. Considering that the pandemic has brought about new opportunities and more uncertainty, understanding the impact of Guanxi orientation on the entry timing of online market has important implications for EBVs intending to compete in the Chinese market.

Aside from Guanxi orientation, entrepreneurial orientation, as a critical element of strategic orientation, has attracted widespread attention from marketing scholars (18). Chinese enterprises adopt entrepreneurial orientation as the guiding principle of business decision-making (19). Entrepreneurial-oriented companies tend to gain competitive advantage by being innovative, risk-taking, and proactive (20). Scholars have pointed out that entrepreneurial orientation can promote market entry (21). However, in the post-pandemic era, the relationship between entrepreneurial orientation and online market entry has been challenged. On the one hand, the essence of entrepreneurship is to identify an unmet need and then provide a product that fulfills such need to the market as quickly as possible (21), which is what the post-pandemic economic market needs. On the other hand, the uncertainty of the policy and health environment brought about by the pandemic has brought about unprecedented risks to entrepreneurs who are developing new business models (22).

The COVID-19 pandemic has posed extraordinary challenges in almost every industry. As a result, entering online markets in search of new market opportunities has become a strategic decision for many companies (23). Previous research has shown that the strengths and weaknesses of a firm's existing resource base jointly determine the timing of market entry (24–26). Despite being widely supported, such studies focus only on the current state of the resource and ignore strategic-level factors beyond the resource. However, strategic-level factors have a tremendous impact on the allocation and investment of corporate resources and play an equally important role in corporate decision-making (27). Therefore, an important research gap is to study the factors influencing firms' market entry timing decisions from a strategic orientation perspective. Furthermore, the outbreak of the epidemic also makes the business environment faced by firms more volatile and uncertain, and firms' strategic decisions are largely subject to changes in the business environment. However, it remains understudied whether firms' market entry decisions are affected by the perceived turbulence of the environment. Therefore, this is the second research gap that this study aims to fill.

In order to fill the above-mentioned research gaps, we develop a research framework investigating the relationship among entrepreneurial orientation, Guanxi orientation, perceived market turbulence, perceived political turbulence, and market entry timing for EBVs, this research aims to achieve two objectives:

1) To test if and how the two strategic orientations, namely, Guanxi orientation and entrepreneurial orientation, influence enterprises' timing of online market entry.
2) To investigate if perceived market turbulence and perceived political turbulence moderate the effects of two types of strategic orientation on online market entry timing.

The present research contributes to the extant literature by integrating various theoretical perspectives: First, it extends the market entry research into the area of E-commerce. Second, building on the social capital theory and resource-based view, this study takes a closer look at how different strategic orientations influence a company's decision on when to enter the online marketplace. Third, this study further identifies the moderating role of perceived environmental turbulence, which is a very important situational variable associated with the COVID-19 pandemic.

## Theoretical background and hypotheses

### Determinants of market entry timing

Market entry can generally be explored from two perspectives, namely, corporate market entry and product market entry. Research from the perspective of corporate focuses on the resources and capabilities (28), whereas research from the perspective of product focuses on the factors related to strategic intention and decision-making (27). On the basis of resource-based theory, previous studies found that the resources and capabilities of an enterprise were the key determinants of its market entry decision and type of entry (24–26). Scholars found that the possession of industry-specific assets determines the timing of entering a certain market (29). Others highlight that firms with different core organizational capabilities, such as manufacturing, market, and R&D capabilities, tend to choose to enter the market at different times (30–32). Moreover, the entry timing of an enterprise largely depends on its dynamic capabilities and varies in different industries (33–35).

Another stream of research focuses on the impact of other factors on entry timing, such as industry or environmental characteristics, the characteristics of the business itself except for resources and capabilities, the behavior of competitors, and enterprise strategy (31, 32, 36, 37), but this kind of research is rare. In terms of industry or environmental characteristics, scholars found that enterprises' market entry timing decisions are influenced by environmental turbulence (36). Moreover, the growth rate of the industry makes a difference in the decision-making of market entry timing. Furthermore, commitment to the market, the size of enterprises, and the degree of diversification of enterprises affect the entry type (32, 38). In addition, the behavior of competitors has an impact on enterprises' entry timing decision (37, 39). When a competitor with the same resources and scale chooses to enter a market, the enterprise will tend to follow and enter the same market.

In summary, both internal factors (e.g., resources, capabilities, and strategies) and external factors (e.g., market, industry environment) are important in determining the entry timing. However, existing studies focus more on the traditional offline market, while research on the online market is quite scarce (40, 41). A considerable number of studies reveal major differences between online and offline markets, of which the role of strategic orientation among the determinants of a company's choice in entering the online market cannot be ignored (6). There is a need for an emerging strand of literature that studies entry timing from the perspective of enterprise strategy (42). Moreover, the outbreak of the COVID-19 pandemic has made the business environment more volatile for firms (23), and existing studies have not examined how the perceived environmental turbulence affects firms' market entry decisions. Therefore, this study will fill these theoretical gaps.

### Strategic orientation and market entry timing

The strategic orientation is a crucial guiding principle for enterprise strategic implementation, decision-making, and target realization, reflecting the strategic direction chosen by an enterprise for superior market performance (43). Following Lee et al. (12)'s view on internal capabilities and external networks, we focused on two types of strategic orientation, namely, Guanxi orientation and entrepreneurial orientation (44, 45).

Small and medium-sized private enterprises have preferential treatment to online markets. China is now in a period of economic transformation and rapid development of e-commerce. Private enterprises are facing high environmental turbulence, whereas the state's institutional support and information support for private enterprises are relatively weak. Under such circumstances, on the one hand, some private enterprises will choose to build their Guanxi networks to obtain support for financing, information, and various important resources, and use these Guanxi networks for effective and efficient economic transactions (46). On the other hand, some enterprises recognize market turbulence as an opportunity, which makes them pay attention to the cultivation of their ability and occupy the market initiative through creative ways (47, 48).

**Figure 1:**
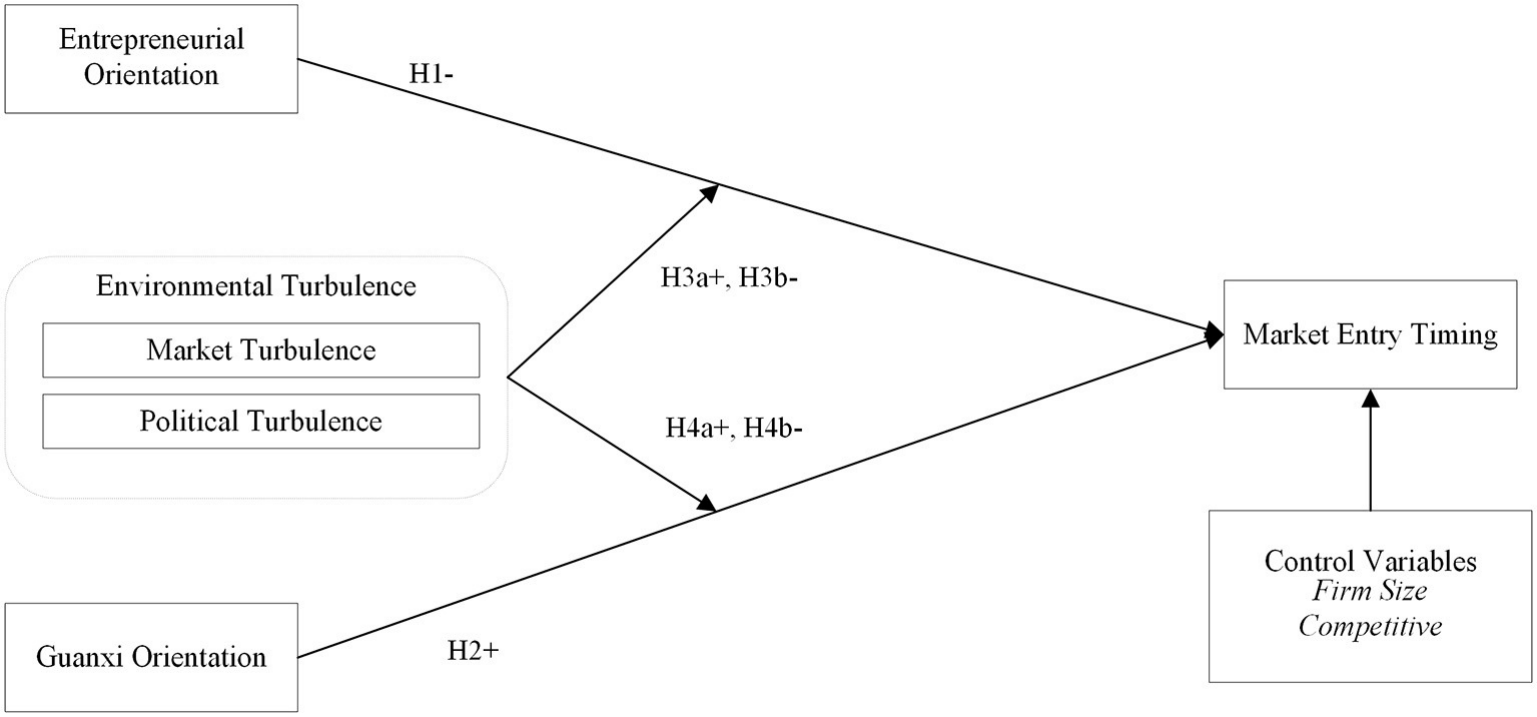
Conceptual model and hypothesized relationships.

The above two mindsets reflect two different strategic orientations of enterprises, namely, Guanxi orientation and entrepreneurial orientation. These two types of orientation are the values and main ideas of enterprises to carry out business activities, which will affect their judgment on market opportunities and their capabilities (49, 50). Moreover, such orientation will guide the allocation of their resources and capabilities. Therefore, we believe that strategic orientation will affect market entry timing (11, 51). Figure 1 depicts our research model including the relationship between two types of strategic orientation and online market entry timing as well as the potential moderating effects of perceived environmental turbulence. We also include firm size and competitive intensity as the control variables to examine the hypothesized relationships (52).

### Entrepreneurial orientation and online market entry timing

Entrepreneurial orientation is part of firm-level strategic orientation, and refers to a firm's strategy-making practices, processes, and behaviors that act entrepreneurially (19). Highly entrepreneurially oriented firms tend to accept actions with uncertain outcomes (21). Some scholars believe that entrepreneurial orientation can enhance enterprises' knowledge capability, thereby affecting their performance in the initial and subsequent periods (53). In addition, highly entrepreneurial-oriented companies are able to learn dynamically through innovation, experimentation, etc., thus mitigating the negative impact of digital platform risks on companies when entering new markets (54). In turn, entrepreneurial orientation may have a strong relationship with the time of market entry. On the one hand, enterprises with high entrepreneurial orientation have higher innovation, risk-taking, and initiative (19). For e-commerce companies in particular, due to the importance of network externalities and first-mover advantage, the timing of EBV's market entry is a critical factor in its success (55). As a result, they may exert more effort to identify opportunities and take early actions to enter the online market (56). On the other hand, firms with high entrepreneurial orientation can aggressively enter a new territory opened by competitors and take corresponding risks (57). For example, they may take the initiative to push their services and products to undeveloped and uncertain markets, encourage enterprises to contact new customers in new markets, and expand the consumer groups of business products (13). Research on international market entry also shows that enterprises with high entrepreneurial orientation are more willing to explore international market opportunities and to enter foreign markets that are completely unfamiliar at an early stage (58). Therefore, we hypothesize that

***H1: The entrepreneurial orientation of enterprises is negatively correlated with the timing of entering the online market: the higher the entrepreneurial orientation is, the earlier they will enter the online market***.

### Guanxi orientation and online market entry timing

Compared with entrepreneurial, Guanxi orientation is a business philosophy built on a relationship management culture that focuses more on strategic goals (6, 16). Previous scholars have elaborated on this concept and developed a robust measurement scale for Guanxi orientation (59). Consistent with previous research, we also define Guanxi orientation as a firm-level strategic orientation, capturing organizational practices and behaviors based on the *mianzi* and *renqing* mechanisms in personal communication, guiding firms to build relationships with stakeholders (59, 60). Guanxi-oriented enterprises are better at maintaining cooperative and win-win relationships with stakeholders and gaining competitive advantage by building effective and robust networks (46, 61). On the one hand, studies have shown that enterprises with higher Guanxi orientation believe that they can quickly gain market competitive advantage by establishing Guanxi, and entry timing is not as important as Guanxi networks (62). Enterprises with high Guanxi orientation highlight the importance of organizational and personal Guanxi in enterprise development (44). The allocation of resources and capabilities of these enterprises focuses on establishing, maintaining, and using Guanxi (63). Such enterprises believe that enterprises can achieve their business goals by improving the Guanxi with the government and business partners, and can also achieve resource acquisition and risk control through these Guanxi (62). On the other hand, enterprises with higher Guanxi orientation have higher risk perception in the market, higher willingness to avoid risks, and are more likely to take risk aversion behaviors (64), thereby delaying entry into the online market. An early online market is often accompanied by higher market risk and technology turbulence. Only when the market is relatively mature, early entrants have cultivated the market, and all kinds of wagers can be controlled will these enterprises enter the online market. To sum up, we propose that:

***H2: The Guanxi orientation of enterprises is positively correlated with the timing of entering the online market: the higher the Guanxi orientation is, the later the firm will enter the online market***.

### Moderating effect of perceived environmental turbulence

Owing to differences in product markets, EBVs are often faced with varying degrees of environmental turbulence. Environmental turbulence includes political turbulence and market turbulence (65). Political turbulence refers to the risks brought about by changes in government policies and regulations, while market turbulence refers to the risk caused by changes in product demand and customer preference, or the emergence of complementary/alternative products. According to the previous enterprise strategy literature, the strategic decision of a company depends on the matching degree between the enterprise and the environment (66, 67). When companies are confronted with political and market turbulence, they must be able to adapt to these turbulence so as to survive and develop (68, 69). The evaluation of their own ability–environment matching varies among enterprises owing to their different strategic orientations. Therefore, when making strategic decisions, they often use different criteria (70).

Entrepreneurial-oriented enterprises are more willing to focus on the innovation of product market when allocating their resources and capabilities. They are inclined to take risks, actively explore market changes, and make adjustments (56). Thus, when market turbulence is high, enterprises with high entrepreneurial orientation will think that their innovation ability can follow and adapt to the changes of market demand, they can use innovative products to meet the changing demand, and stand out in the competition, thereby forming a competitive advantage (71). However, when allocating resources and capabilities, enterprises with high entrepreneurial orientation cannot respond to policy changes effectively, because difficulties brought about by policy changes are difficult to overcome through product innovation (72). Therefore, when political turbulence is high, enterprises with high entrepreneurial orientation will consider delaying their entry into the market. To sum up, we propose the following hypotheses:

***H3a: The higher the market turbulence is, the stronger the negative impact of entrepreneurial orientation has on online market entry timing***.
***H3b: The higher the political turbulence is, the weaker the negative impact of entrepreneurial orientation has on online market entry timing***.

Enterprises with high Guanxi orientation tend to use resources and capabilities to establish and maintain Guanxi (46). Through Guanxi, enterprises can easily obtain business information and financial/non-financial support provided by the government (73). On the one hand, as a higher Guanxi orientation makes enterprises more sensitive to market risk and cost (62, 74), Guanxi-oriented enterprises are more willing to delay market entry. In addition, highly Guanxi-oriented enterprises are also reluctant to respond to market turbulence through high-risk and high-cost innovation (75). On the other hand, when there is political turbulence, enterprises with higher Guanxi orientation are more willing to enter the market early. They may consider themselves more able to take risks from political turbulence and even benefit from such turbulence (76). Owing to the close Guanxi with the government, enterprises with higher Guanxi orientation have more advantages and protection in obtaining the scarce information provided by the government (6, 77). They can use this information to make strategic deployments in advance and win the first chance in the market competition (78).

To sum up, we make the following assumptions:

***H4a: The higher the perceived market turbulence is, the stronger the positive impact of Guanxi orientation has on online market entry timing***.
***H4b: The higher the perceived political turbulence is, the weaker the positive impact of Guanxi orientation has on online market entry timing***.

## Methodology

### Data collection

To test the hypotheses in the research framework, we surveyed EBVs that entered the Chinese online market in the past 10 years. Over 10,000 active EBVs' contact information was obtained through an electronic business platform. This database contains a large number of enterprises and a full range of enterprise types, including state-owned enterprises, private enterprises, foreign-funded enterprises, etc., so our sample is highly representative (79). All the vendors doing business on the electronic business platform were established after 2008. We emailed 1,134 randomly selected EBVs from this list and asked them to participate in our survey. Within 1 month, we received 377 EBVs agreements of participating in this research. Next, we emailed the survey linkage to the 377 companies and prompted executives to complete the questionnaire in person. To alleviate the concern about the misuse of the collected information, we emphasized that all the data is only for academic research, and no information will be disclosed. We received 174 valid responses in a three-month period, with an acceptable response rate of 46.15%. Table 1 presents our sample characteristics.

**Table 1:** Sample characteristics.

**Characteristics**	**N**	**%**
**Employees/Firm size**		
30 or less	97	55.75%
30–100	42	24.14%
100–200	19	10.92%
200 or more	16	9.20%
**Sales revenue (2018, in US$)**		
1.46 million or less	95	54.60%
1.46–4.37 million	41	23.56%
4.37–7.28 million	23	13.22%
7.28 million and up	15	8.62%
**Headquarter location**		
South-East coastal areas	102	58.62%
Inland regions	72	41.38%
**Industry type**		
Fashion and apparel	71	40.80%
Nutrition and food services	26	14.94%
Cosmetics and healthcare	29	16.67%
Household and cleaning supply	18	10.34%
Home furnishing and home decor	16	9.20%
Electronics and information technology	14	8.05%
**Education (chief executive)**		
Less than or high school graduate	41	23.56%
Some college	68	39.08%
Bachelor's degree	55	31.61%
Graduate degree	10	5.75%
**Gender (chief executive)**		
Male	132	75.86%
Female	42	24.14%
**Age (chief executive)**		
18–25 years	18	10.34%
26–30 years	72	41.38%
31–40 years	49	28.16%
41–50 years	35	20.11%
**Entry timing**		
Market pioneers	41	23.56%
Early followers	80	45.98%
Late entrants	53	30.46%

In order to test the threat of non-response bias, we performed a t-test between key variables in early and late respondents. We found non-significant difference (*p* >0.05) between the two groups. Thus, there is no evidence of non-response bias.

### Constructs and measurement

We adapted all our measurement scales from the extant literature. Except for the online market entry timing measures, all constructs are measured on a five-point Liker scale, with one equals strongly disagree and seven equals strongly agree. Following Niu et al. (80), we measured online market entry timing with three continuous categories: market pioneers, early followers, and late entrants. Table 2 shows all scale items (except for the online market entry timing).

**Table 2:** Assessment of reflective measures.

**Measure**	**Items**	**Factor loading**	**Cronbach's α**	**Composite reliability**	**AVE**
Guanxi Orientation (GXO)	Birds of a feather flock together (GXO1)	0.78	0.882	0.902	0.609
	Business intercourse entails giving face (mianzi) to your partners (GXO2)	0.84			
	Don't suspect your business partner, because trust begets trust (GXO3)	0.586			
	One tree doesn't make a forest (GXO4)	0.812			
	Give a hand when your friend is in adversity (GXO5)	0.835			
	Business dealings entail reciprocity (GXO6)	0.801			
Entrepreneurial orientation (EO)	When it comes to problem solving, we value creative new solutions more than the solutions of conventional wisdom. (EO1)	0.864	0.878	0.901	0.695
	Our top managers encourage the development of innovative marketing strategies, knowing well that some will fail. (EO2)	0.769			
	We firmly believe that a change in market creates a positive opportunity for us. (EO3)	0.869			
	We tend to talk more about opportunities rather than problems. (EO4)	0.831			
Market turbulence (MATUR)	In our industry, customers' product preferences change quite a bit over time. (MATUR1)	0.754	0.783	0.856	0.601
	Our customers tend to look for new products/services all the time. (MATUR2)	0.809			
	We are witnessing demand for our products and services from customers who never bought them before. (MATUR3)	0.634			
	New customers tend to have product-related needs that are different from those of our existing customers. (MATUR4)	0.884			
Political turbulence (POTUR)	In our industry, the authorities act in a way that cause us great uncertainty. (POTUR1)	0.911	0.872	0.92	0.794
	It is hard to predict the impact of the policy changes on the market situation in our industry. (POTUR2)	0.903			
	In our industry, it is hard to predict policy changes. (POTUR3)	0.861			
Competitive intensity (COINT)	Competition in our industry is cutthroat. (COINT1)	0.781	0.770	0.837	0.510
	Anything that one competitor can offer, others can match easily. (COINT2)	0.723			
	Price competition is a hallmark of our industry. (COINT3)	0.566			
	There are too many similar products in the market; it is difficult to differentiate our products/services. (COINT4)	0.741			
	One hears of a new competitive move almost every day. (COINT5)	0.741			

A bilingual professor translated the original scales from English into Chinese. A separate bilingual translator carried out a backwards translation for authentication purposes (81). We also asked eight graduate business students and two linguists to evaluate the cross-cultural measurement equivalence in the Chinese and English versions (10). We then pre-tested the Chinese-version questionnaire on 32 Chinese companies. Based on the pre-test results, we refined the questionnaire before sending it out.

To evaluate the threat of common method bias (CMB), we use the method suggested by MacKenzie and Podsakoff (82). Moreover, we utilized collinearity VIF in SmartPLS to assess CMB by connecting all the variables to a single variable. There is method bias if the VIF is >3.3 at the factor level (83). The highest VIF in our study is 2.69, so we did not violate the assumptions of common method bias. Therefore, CMB is not an issue in the model.

## Result

### Reliability and validity of measures

SmartPLS 3.0 was used to assess the reliability and validity of the constructs in this study. In Table 2, all composite reliability and Cronbach's alpha are above the minimum 0.7 for internal consistency reliability (84). In addition, the majority of the factor loadings exceed the suggested value 0.70 (85). Only three factor loadings are between 0.40 and 0.70, below the threshold value. However, we retained these items as the composite reliability value of each related construct does not increase after deleting these items (85). Therefore, our reflective measures show acceptable indicator reliability. Lastly, average variance extracted (AVE) values are all above 0.50, which indicates convergent validity is met. Table 3 indicates a good discriminant validity, as the square root of AVEs is larger than the correlation of latent constructs (86).

**Table 3:** Results of discriminant analysis.

**Latent variables**	**CI**	**EO**	**GXO**	**MATUR**	**POTUR**
Competitive intensity	(0.714)				
Entrepreneurial orientation	0.163	(0.834)			
Guanxi orientation	0.325	0.084	(0.781)		
Market turbulence	0.333	0.282	0.094	(0.775)	
Political turbulence	0.191	0.152	0.187	0.089	(0.891)

Diagonal elements are the square root of AVEs. The off-diagonal elements are the correlations among latent variables.

### Analysis of the structural model

**Figure 2:**
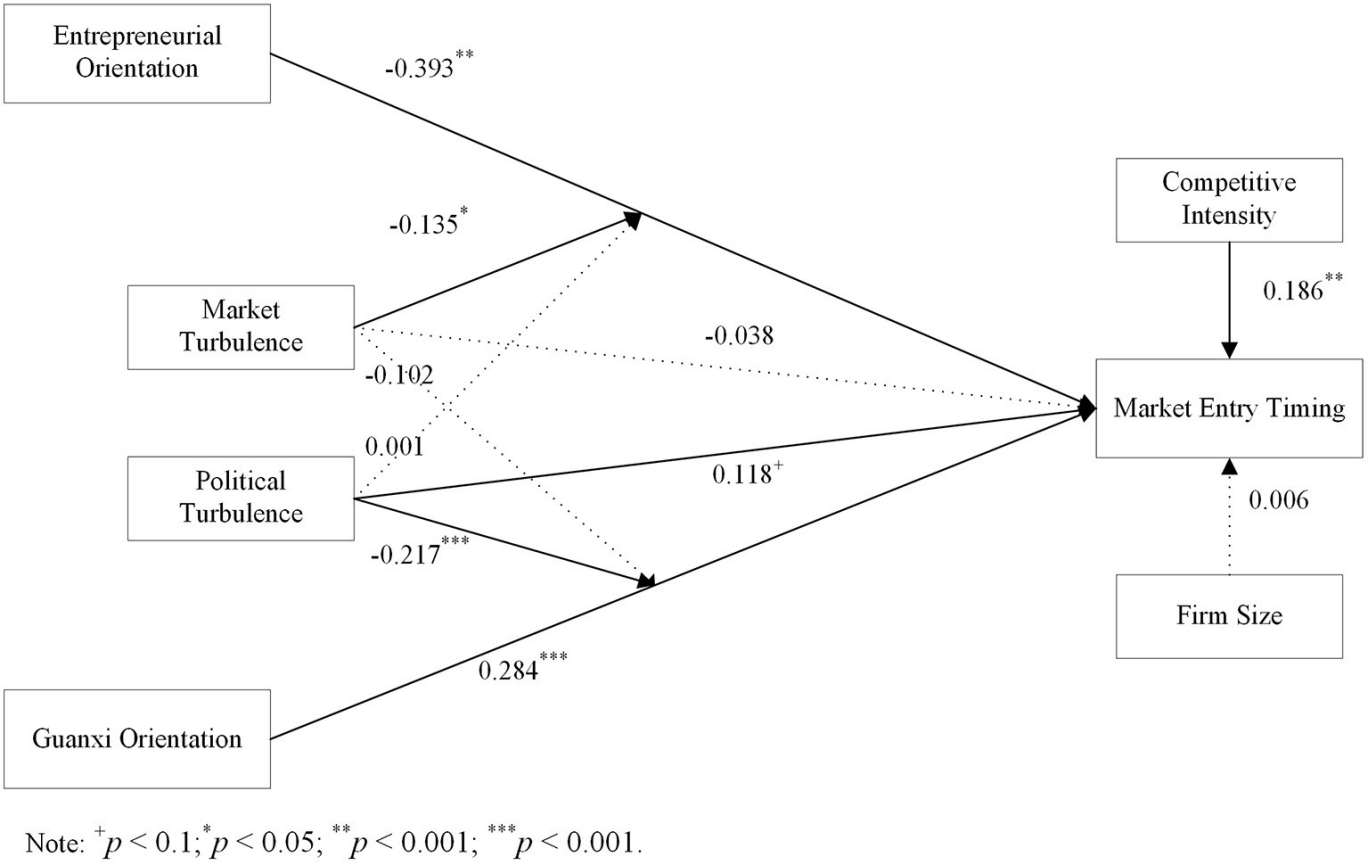
Results of the structural model.

Figure 2 presents the results of the PLS model. The model explains 43.7% of the variance, indicating its good predictive power. In addition, the standardized root mean square residual (SRMR) was 0.074 below 0.08, indicating a goodness fit of our research model (87). This study conducts the bootstrap analysis with 5,000 samples to generate the standard errors and t values (88, 89). We employed partial least squares (PLS) regression to test all our hypotheses for three reasons: (1) PLS can handle reflective and formative measurements simultaneously; (2) PLS can provide robust results when dealing with a relatively small sample; (3) PLS is suitable for running predictive models (88). Before we run the structural model in SmartPLS software, we assess the collinearity threat using the summated scores of the latent variables. The largest variance inflation factor (VIF) value of exogenous variables was 2.288, below 5, indicating that the collinearity problem was not a threat in this study (90).

In terms of the control variables, we found that firm size (β = 0.006, *p* > 0.50) has no impact on online market entry timing. However, competitive intensity is positively related to online market timing (β = 0.186, *p* < 0.01). Thus, when the competition is intensive, companies tend to enter the online market later. After controlling for the effects of firm size and competitive intensity, entrepreneurial orientation (β = −0.393, *p* < 0.001) is negatively related to online market entry timing. Thus, H1 is supported. Guanxi orientation (β = 0.284, *p* < 0.001) is positively related to online market entry timing, supporting H2. Furthermore, political turbulence has a marginal significant impact on online market entry timing (β = 0.118, *p* < 0.10). However, market turbulence has no significant effect on online market entry timing (β = −0.038, *p* > 0.50).

In terms of the moderation effects, the negative impact of entrepreneurial orientation on online market entry timing is strengthened when perceived market turbulence is high (β = −0.135, *p* < 0.005). However, we do not observe the moderation effect of perceived political turbulence on the relationship between entrepreneurial orientation and online market entry timing (β = 0.001, p > 0.10). Therefore, H3a is supported, but H3b is not supported.

Furthermore, the positive impact of Guanxi orientation on online market entry timing is weakened when perceived political turbulence is high (β = −0.217, *p* < 0.001). However, we do not observe the moderation effect of perceived market turbulence on the relationship between Guanxi orientation and online market entry timing (β = −0.102, p > 0.10). Therefore, H4b is supported, but H4a is not supported.

## Discussion

### Discussion of findings

Our results provide valuable insights into how strategic orientations impact online market entry timing in the online market after the COVID-19 pandemic, with China as an example. First, strategic orientation has a significant impact on entry timing decisions, however, the effects of entrepreneurial and Guanxi orientation are different. Enterprises with high traditional Chinese Guanxi orientation are more willing to enter the online market in a late stage, whereas enterprises with high entrepreneurial orientation are more inclined to be the early entrants. These two strategic orientations have completely opposite impacts on online market entry timing, which is an interesting reflection on the different effects of Chinese and Western management mindsets on business decision-making (91). The reason for this difference lies in the different ways that enterprises with high Guanxi orientation and those with high entrepreneurial orientation judge the match of their own resources and capabilities with market opportunities (16). Companies with high Guanxi orientation believe that they can utilize their Guanxi resources to obtain sustainable competitive advantage or late-mover advantage after the market becomes mature (6), whereas companies with high entrepreneurial orientation are more confident that they can quickly seize the early market opportunities and establish technology advantage and customer-switching cost advantage (19).

In addition, different types of EBVs have different applicability to strategic orientation. For instance, by offering clients healthy and convenient healthcare options, medical online platforms (e.g., telemedicine) have become particularly useful in the context of the COVID-19 pandemic. Due to the high risk and uncertainty of medical online platforms and the high costs of entrepreneurial orientation implementation (92, 93), a good Guanxi orientation can help healthcare companies better access online platforms, reduce costs and improve efficiency by achieving business goals through management ties with partners. On the one hand, medical service products are related to public health and are subject to many policy constraints, which require close communication with government and regulatory agencies; on the other hand, medical products and services are highly technical and specialized, which require stable relationships with business partners. Therefore, we argue that Guanxi orientation is more appropriate for healthcare providers that enter medical online platforms.

Second, the impact of strategic orientation on online market entry timing depends on the perception of external environmental turbulence, especially in the era after the COVID-19 pandemic when environmental turbulence is at its peak. Specifically, companies with high entrepreneurial orientation tend to enter the market quickly when they perceive that market turbulence is high in the industry. When political turbulence is high, the impact of Guanxi orientation on online market entry timing becomes weaker. These two findings are consistent with our hypothesis. According to resource dependence theory, enterprises with different strategic orientations view opportunities and threats from turbulent environments from distinct perspectives (25). When the external market environment becomes turbulent, the needs of users or/and groups of users undergo drastic changes (94). Companies with high entrepreneurial orientation will consider this kind of turbulence an opportunity and conducive to their development rather than a threat, because they are confident to insight and meet the needs of users (95). Therefore, market turbulence will promote enterprises with high entrepreneurial orientation to accelerate their entry into the online market. When the external political environment becomes turbulent, the industry policy may change or become uncertain (96). Companies with high Guanxi orientation will regard this kind of turbulent policy as a relative competitive advantage for them (63). Enterprises with high Guanxi orientation tend to invest more in Guanxi, so they are able to take advantage of this Guanxi and take the lead in making profits or avoiding risks in turbulent policy environments (63).

However, two hypotheses (H3b and H4a) have not been confirmed yet in our study. Political turbulence does not moderate the effect of entrepreneurial orientation on online market entry timing. Companies with high entrepreneurial orientation will not delay their entry into the e-market under a turbulent policy environment. The reason for the inconsistency may be that the ultra-high growth of China's e-market in recent years encourages enterprises with high entrepreneurial orientation to pay more attention to the enormous opportunities presented in the market, while ignoring the potential threat of changes in the political environment (97, 98). Given the insensitivity of entrepreneurial-oriented firms to external policy changes, the impact of entrepreneurial orientation on entry timing does not depend on political turbulence. Similarly, market turbulence does not adjust the impact of Guanxi orientation on online market entry timing. Enterprises with higher Guanxi orientation will not delay online market entry timing due to drastic changes in market demand. This may be due to the fact that market turbulence does not pose a great threat to enterprises with a higher Guanxi orientation. For highly Guanxi-oriented enterprises, the resources to deal with market turbulence are easily accessible. Therefore, market turbulence plays a less prominent role in this scenario.

### Contributions

This study's findings provide meaningful contributions to the literature and practitioners in three aspects. First, most previous studies see resources and capabilities as the determining factors influencing the timing of market/online market entry (28, 99). Our research confirms that strategic orientation also has a significant effect on online market entry timing. Strategic orientation is a basic factor that directly affects the allocation of resources and capabilities (100). Moreover, the Guanxi orientation based on Chinese culture and the entrepreneurial orientation based on Western culture have different effects on the online market entry timing, which provides new insights for cross-cultural research. In addition, this result highlights the importance of enterprises' understanding in their strategic orientation as well as the scope of application of different strategic orientations. For example, in the regions (e.g., China) and areas (e.g., healthcare) where Guanxi orientation is more important, companies should consider delaying market entry.

Second, our research object is the online market entry behavior of EBVs, whereas the majority of the previous research is offline market entry behavior. E-commerce platform and offline market display great differences in information richness, information symmetry, and information acquisition ability of buyers (101, 102). Traditional research conclusions on offline-to-online market entry are not necessarily applicable to the e-commerce platform (103). For example, for EBVs, the uncertainty of the market is very different from the offline market due to the openness of the platform and the widespread use of information technology. Accordingly, the approaches to maintaining their customers and the models of value co-creation are also different (104). In turn, the allocation of resources and capabilities in determining market entry time into the online market will vary, making the conclusion of this study of higher pertinence and timeliness for e-commerce platforms.

Finally, from the perspective of perceived environmental turbulence, the study explored the boundary of the impact of strategic orientation on online market entry timing and refined the moderating effects of two types of perceived environmental turbulence (including perceived market turbulence and perceived political turbulence). An interesting finding is that perceived market turbulence only moderates the effects of entrepreneurial orientation on online market entry timing, whereas perceived political turbulence only moderates the relationship between Guanxi orientation and online market entry timing. These findings indicate that the influence of entrepreneurial orientation on entry timing depends only on market changes, whereas the impact of relationship orientation depends only on political turbulence. As the impact of political turbulence is more unpredictable, it may enhance companies' perception of market uncertainties (95). Moreover, as far as market turbulence is concerned, its effect on the market behavior of companies is complex and varies from stage to stage (105). Therefore, companies might endeavor to effectively identify and evaluate different types of environmental turbulence when making market decisions. This kind of detailed research offers an in-depth insight for online market entry research.

### Limitations and future research

This study still has several limitations. First, limited by sample size, we did not classify EBVs by industry type to study the potential differences. Future research could investigate if the findings remain consistent among various industries. For instance, do the effects of two types of strategic orientation (Guanxi and entrepreneurial orientation) on online market entry timing differ across high-tech and fashion companies? To provide insights in greater depth, future research could investigate the distinctive characteristics associated with a given industry type.

Second, the current research strictly focused on two kinds of firm-level strategic orientation: Guanxi and entrepreneurial orientation in China's online market. What is the relative importance of entrepreneurial and Guanxi orientation among other strategic orientations? Future research could consider the inclusion of other types of strategic orientation, such as competition or customer orientation, in the study of online market entry timing in the online market. Third, many online companies also manage offline business operations. However, how Guanxi and entrepreneurial orientation affect online and offline business differently was not considered. Thus, future research could investigate this difference in hypotheses development as well as in the data collection process.

Finally, the impact of the COVID-19 pandemic on the online market is multifaceted. Especially, the continued disruption to consumer behavior and supply chains has prompted necessary changes in corporate business activities. This research particularly investigates the relationship between strategic orientation and market entry. On the one hand, since more companies are moving their businesses online, future research could focus on changes in companies' online business models. On the other hand, the epidemic has prompted companies to use various emerging technologies to connect with consumers, such as contactless payment and social media, to enhance customer experience. Future research could explore the impact and application of emerging technologies on online platforms.

## Data availability statement

The raw data supporting the conclusions of this article will be made available by the authors, without undue reservation.

## Author contributions

HM: conceptualization, methodology, and funding acquisition. CH: formal analysis and writing—original draft. XH: resources, writing—review and editing, and investigation. BW: writing—review and editing and writing—original draft. ZC: funding acquisition, formal analysis, conceptualization, and writing—original draft. LZ: writing—original draft, funding acquisition, and conceptualization. All authors contributed to the article and approved the submitted version.

## Funding

This work was supported by the National Natural Science Foundation (NSFC) Programs of China (72061005, 71902126), Guizhou Provincial Science and Technology Projects [(2020)1Y285, (2019)5103], Guizhou Youth Science and Technology Talent Growth Project [KY(2021)125], Startup Foundation for Distinguished Scholars of Guizhou University of Finance and Economics (2019YJ065), Shanghai Philosophy and Social Science Planning Project (2019EGL020), the Soft Science Research Project of Shanghai Science and Technology Innovation Action Plan (22692197800), and Project of Humanities and Social Science of Jiangsu Province (20GLC005).

## Conflict of interest

The authors declare that the research was conducted in the absence of any commercial or financial relationships that could be construed as a potential conflict of interest.

## Publisher's note

All claims expressed in this article are solely those of the authors and do not necessarily represent those of their affiliated organizations, or those of the publisher, the editors and the reviewers. Any product that may be evaluated in this article, or claim that may be made by its manufacturer, is not guaranteed or endorsed by the publisher.
